# Acute *Coxiella burnetii* infection presenting as sepsis with multisystem involvement in an immunosuppressed patient: a case report

**DOI:** 10.3389/fmed.2026.1899347

**Published:** 2026-08-24

**Authors:** Yaozong Xia, Lin Fu, Xiu Cao, Xuxiang Zheng, Bin Nie

**Affiliations:** 1 Department of Laboratory Medicine, The Second People’s Hospital of Yibin, Yibin, China; 2 Clinical Research and Translational Center, The Second People’s Hospital of Yibin-West China Yibin Hospital, Sichuan University, Yibin, China

**Keywords:** *Coxiella burnetii*, immunosuppressed, metagenomic next-generation sequencing, Q fever, sepsis

## Abstract

Q fever, caused by *Coxiella burnetii*, rarely presents as severe disseminated disease, and timely diagnosis can be difficult because clinical manifestations are nonspecific and routine microbiological tests are often unrevealing. We report a 68-year-old man with rheumatoid arthritis receiving long-term immunosuppressive therapy who presented with persistent unexplained fever, pancytopenia, hepatic dysfunction, polyserosal effusions, and sepsis with multisystem involvement, without a clear epidemiological exposure history. Blood cultures and routine respiratory pathogen testing were negative. Peripheral-blood metagenomic next-generation sequencing (mNGS) detected *C. burnetii* nucleic acid sequences, and subsequent antibody testing and targeted qPCR supported the diagnosis of acute Q fever with disseminated manifestations. Doxycycline-based targeted therapy was followed by defervescence and marked clinical and laboratory improvement. This case suggests that, in selected immunocompromised patients with severe infection and persistently negative conventional investigations, mNGS may serve as an adjunctive tool to facilitate timely pathogen identification and guide targeted antimicrobial therapy.

## Introduction

*Coxiella burnetii* is an obligate intracellular Gram-negative coccobacillus and the causative agent of Q fever, a zoonosis usually acquired through inhalation of contaminated aerosols (1–3). Exposure may occur through contact with infected animals, particularly cattle and sheep, but a clear history of direct animal contact is not required for diagnosis (1). Acute Q fever has a broad and nonspecific clinical spectrum, ranging from self-limited febrile illness to pneumonia or hepatitis (1, 3). Severe presentations with sepsis and multisystem involvement are uncommon and can be difficult to recognize early (4–6). Chronic Q fever most commonly manifests as infective endocarditis and requires careful follow-up in at-risk patients (7–9).

The diagnosis of Q fever is complicated by the intracellular biology of *C. burnetii* and the limitations of conventional testing. The organism does not grow readily on routine culture media, and blood cultures are typically negative (1, 8). Serological testing, including phase I and phase II antibody detection, remains the diagnostic mainstay. However, results may be delayed, and false-negative findings can occur early in the disease course (10). In critically ill patients with sepsis of unknown origin, multisystem involvement, and negative routine microbiological tests, these limitations may delay targeted treatment (10, 11).

Metagenomic next-generation sequencing (mNGS) is an unbiased, culture-independent diagnostic approach that enables broad-range detection of bacteria, viruses, fungi, and parasites directly from clinical samples (12, 13). It has shown utility in identifying rare, unexpected, or difficult-to-culture organisms in severe or diagnostically challenging infections (14–17). Here, we report a case of acute *C. burnetii* infection in an immunosuppressed host without a clear epidemiological exposure history, presenting as sepsis with multisystem involvement and a concurrent cerebrovascular event. Conventional microbiological investigations were unrevealing, whereas peripheral-blood mNGS detected *C. burnetii* nucleic acid, prompting doxycycline-based targeted therapy and subsequent clinical improvement. This case emphasizes the need to consider Q fever in selected culture-negative severe infections and illustrates the adjunctive diagnostic value of mNGS in complex immunosuppressed hosts.

## Case presentation

### Patient information

A 68-year-old man was admitted to the Department of Rheumatology and Immunology on June 8, 2025, with a one-week history of progressive gait instability and dizziness. He had a five-year history of rheumatoid arthritis (RA) and had been receiving long-term immunosuppressive therapy with methotrexate (12.5 mg/week) and leflunomide (20 mg/day), with adequate disease control before admission. During the 5-year medication period, complete laboratory tests including blood routine and liver and kidney function were conducted at each follow-up visit, and no abnormal liver enzyme levels were found.

### Presenting symptoms and medical history

One week before admission, he developed progressive unsteadiness while standing and walking, accompanied by dizziness and without an identifiable trigger. He had no dysarthria or limb weakness. Cranial CT at an outside hospital showed cerebral atrophy with white matter changes, and symptomatic treatment did not improve his symptoms. His appetite decreased after symptom onset, with mild weight loss, whereas mental status and sleep were largely preserved.

His history included 40 years of smoking, which he had stopped one year earlier, and occasional alcohol use. He was a chronic hepatitis B virus (HBV) carrier and was receiving entecavir (0.5 mg/day). During the 5-year medication period, complete laboratory tests including blood routine and liver and kidney function tests were conducted at each follow-up visit, and no abnormal liver enzyme levels were found. He denied hypertension, diabetes mellitus, cardiac disease, recent travel, or animal contact.

### Findings on admission

On admission, his vital signs were stable: temperature 36.2 °C, pulse 90 beats/min, respiratory rate 20 breaths/min, and blood pressure 124/73 mmHg. Physical examination revealed mild pallor, with no definite localizing neurological signs and no joint swelling or tenderness.

Initial laboratory tests showed pancytopenia and systemic inflammation: white blood cell count (WBC) 2.18 × 10^9^/L (reference 3.5–9.5 × 10^9^/L), absolute neutrophil count (ANC) 1.32 × 10^9^/L (reference 1.8–6.3 × 10^9^/L), hemoglobin (Hb) 98 g/L (reference 130–175 g/L), platelet count (PLT) 88 × 10^9^/L (reference 125–350 × 10^9^/L), C-reactive protein (CRP) 54.75 mg/L (reference <10 mg/L), and procalcitonin (PCT) 1.01 ng/mL (reference <0.05 ng/mL). Hepatic involvement was suggested by elevated alanine aminotransferase (ALT) 79.90 U/L (reference 9–50 U/L) and aspartate aminotransferase (AST) 100.00 U/L (reference 15–40 U/L), together with hypoalbuminemia (albumin 33.90 g/L; reference 40–55 g/L).

CT angiography of the head and neck revealed extensive occlusion of the right middle cerebral artery at the M1–M2 segment and patchy hypodensity in the right temporal lobe, consistent with cerebral infarction. Methotrexate and leflunomide were withheld. Initial management focused on the cerebrovascular event and included aspirin (100 mg/day), rosuvastatin (10 mg nightly), and supportive neuroprotective and hepatoprotective care.

### Disease evolution

**Figure 1:**
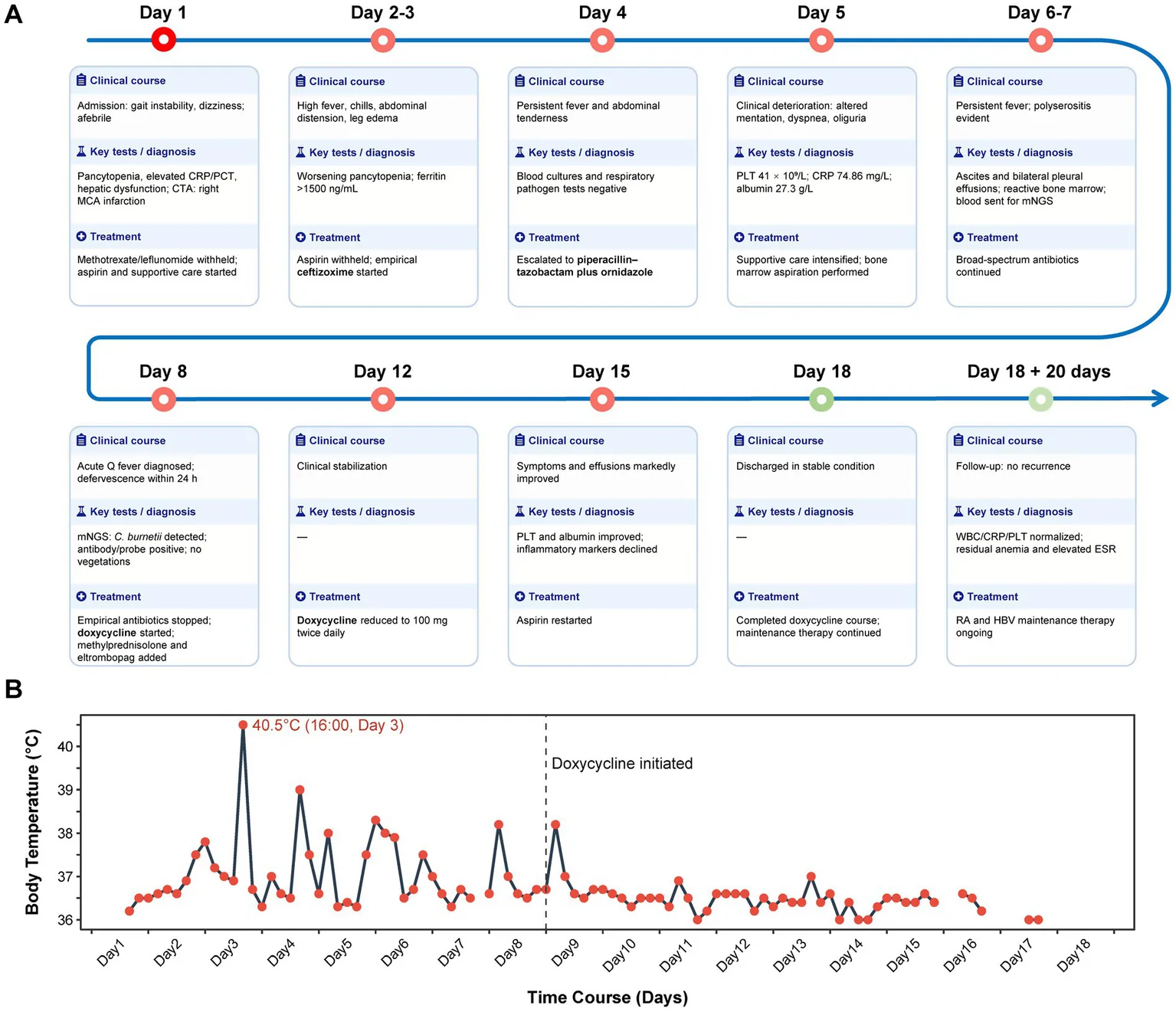
Clinical timeline and body temperature trend of the patient during hospitalization. **(A)** Timeline of the patient’s clinical course from admission to follow-up, including major symptoms, key diagnostic findings, and treatment adjustments. **(B)** Body temperature curve during hospitalization. Recurrent high fever developed after admission, with a peak temperature of 40.5 °C. Body temperature normalized within 24 h after initiation of doxycycline on hospital day 8 and remained stable thereafter. CRP, C-reactive protein; CTA, computed tomography angiography; ESR, erythrocyte sedimentation rate; HBV, hepatitis B virus; MCA, middle cerebral artery; mNGS, metagenomic next-generation sequencing; PLT, platelet count; RA, rheumatoid arthritis.

His condition deteriorated rapidly after admission (Figure 1A). On hospital days 2–3, he developed recurrent high fever, with a peak temperature of 40.5 °C (Figure 1B), accompanied by chills and rigors, followed by progressive abdominal distension, abdominal pain, and bilateral lower-limb edema. Repeat laboratory tests showed worsening pancytopenia (WBC 2.57 × 10^9^/L, Hb 87.0 g/L, PLT 66 × 10^9^/L), persistently elevated inflammatory markers (CRP 61.16 mg/L), and markedly elevated ferritin (>1,500 ng/mL; reference 30–400 ng/mL). Anti-cyclic citrullinated peptide antibody (86.20 U/mL; reference <17 U/mL) and rheumatoid factor (34.3 IU/mL; reference <20 IU/mL) were consistent with the established RA diagnosis. Infection was considered the leading etiology. Aspirin was withheld because of thrombocytopenia, and empirical antimicrobial therapy with ceftizoxime (2 g intravenously every 8 h) was initiated. Blood cultures and nucleic acid testing for common respiratory pathogens, including influenza A and B viruses, respiratory syncytial virus, *Mycoplasma pneumoniae*, and SARS-CoV-2, were obtained.

On hospital day 4, fever persisted despite ceftizoxime. Abdominal tenderness and equivocal shifting dullness were noted, and lower-limb edema persisted. Blood cultures and respiratory pathogen tests were negative. Antimicrobial therapy was escalated to piperacillin-tazobactam (10 g intravenously every 8 h) plus ornidazole (0.4 g/day).

On hospital day 5, the patient deteriorated further. Fever with chills and rigors persisted. Breath sounds were reduced at both lung bases, the abdomen was distended with marked tenderness, and abdominal girth had increased. He developed altered mentation, nausea, reduced appetite, exertional dyspnea, and oliguria. Repeat laboratory testing demonstrated worsening thrombocytopenia, systemic inflammation, hepatic dysfunction, and hypoalbuminemia, with PLT 41 × 10^9^/L, CRP 74.86 mg/L, ALT 58.60 U/L, AST 74.50 U/L, albumin 27.30 g/L, and prealbumin 34.50 mg/L, accompanied by mild hyponatremia and hypocalcemia. Given the sustained fever, progressive thrombocytopenia, worsening hypoalbuminemia, rapidly increasing ascites, oliguria, and evolving serosal effusions, he was considered at risk of progression to septic shock and multisystem dysfunction. Antimicrobial therapy was intensified, and intravenous albumin, supplemental oxygen, and close monitoring were instituted. Blood cultures were repeated, and bone marrow aspiration with cytological and flow cytometric analysis was performed to evaluate persistent pancytopenia.

**Figure 2:**
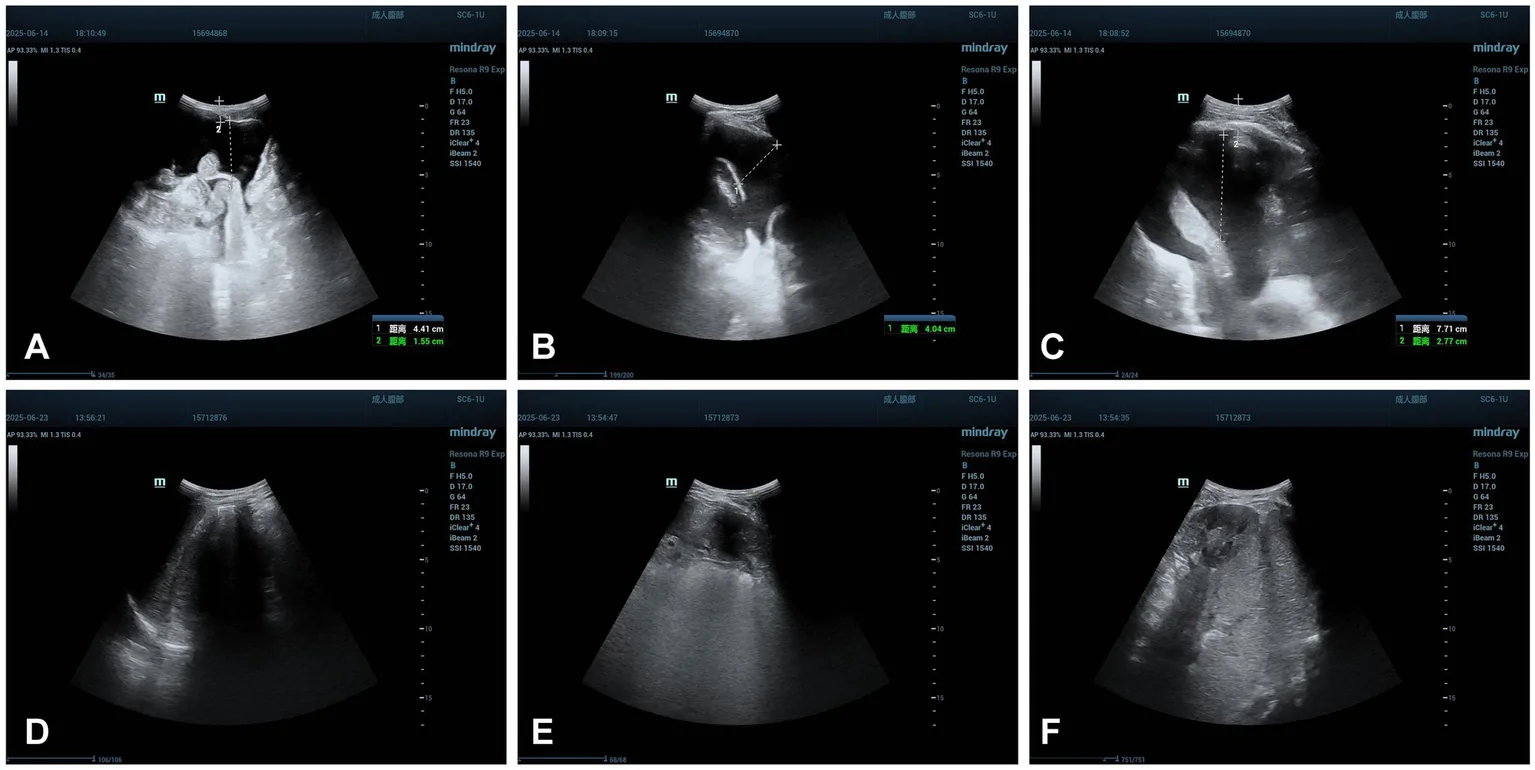
Ultrasonographic changes in abdominal and pleural effusions during hospitalization. **(A)** Abdominal ultrasonography on hospital day 7 showed ascites. **(B,C)** Thoracic ultrasonography on the same day showed left- and right-sided pleural effusions, respectively. **(D)** Follow-up abdominal ultrasonography on hospital day 15 showed marked resolution of ascites. **(E,F)** Follow-up thoracic ultrasonography on hospital day 15 showed no obvious residual pleural effusion bilaterally.

On hospital days 6–7, fever and rigors persisted, although his overall condition did not deteriorate further. Bedside ultrasound confirmed ascites and bilateral pleural effusions (Figures 2A–C). Bone marrow examination showed no evidence of hematologic malignancy, and the findings favored a reactive marrow response to infection or systemic inflammation. Because fever, pancytopenia, polyserosal effusions, lack of response to empirical antimicrobial therapy, and negative conventional microbiological testing persisted, peripheral blood was submitted for mNGS on hospital day 7. The mNGS workflow is detailed in the Supplementary material. Thoracentesis was performed concurrently, and pleural fluid was sent for routine analysis, biochemistry, acid-fast bacilli smear, and *Mycobacterium tuberculosis* DNA testing.

### Application and findings of mNGS

**Figure 3:**
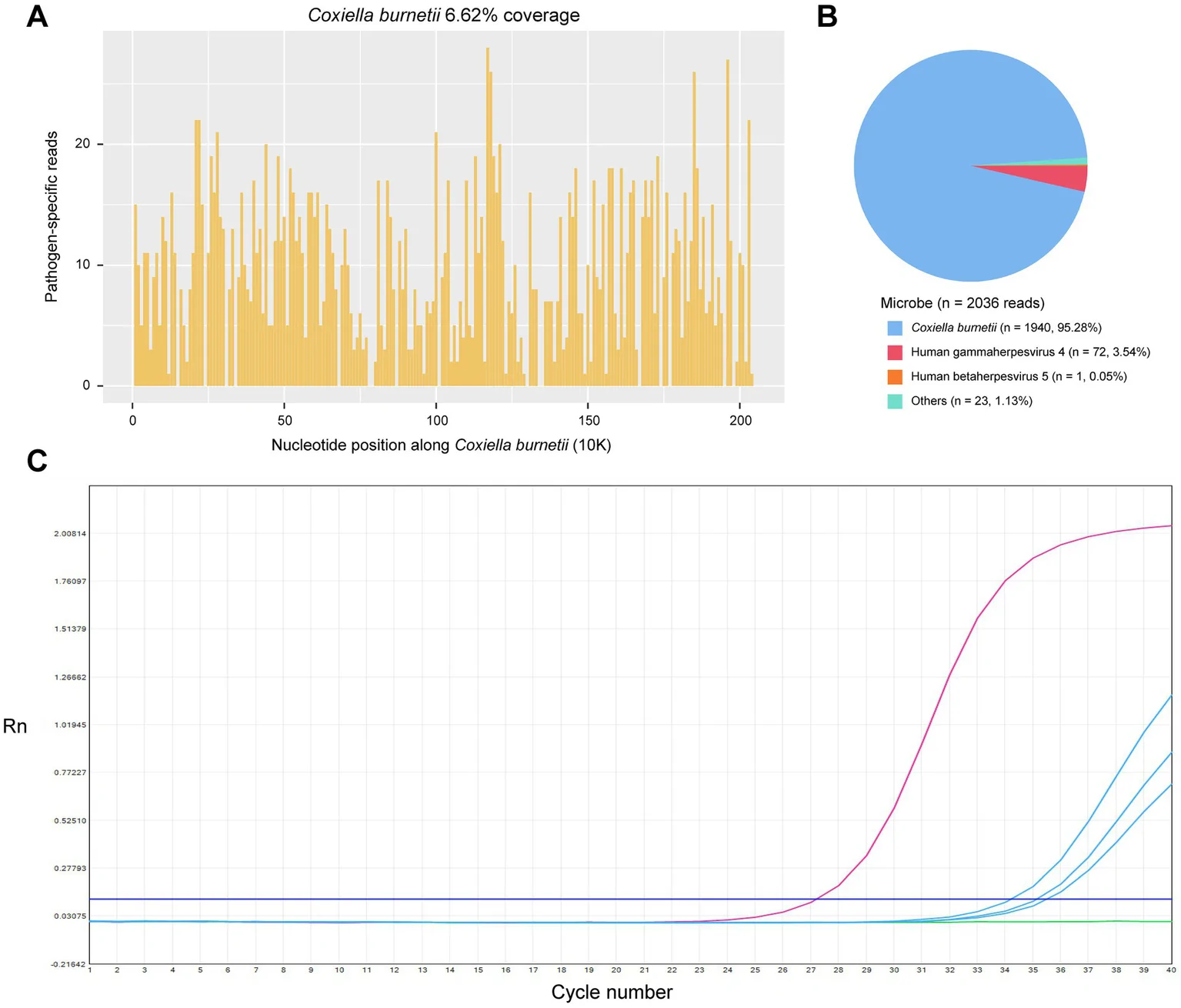
Identification and confirmation of *Coxiella burnetii* infection by mNGS and targeted qPCR. **(A)** Distribution of *Coxiella burnetii*-specific reads across the reference genome detected by peripheral-blood mNGS, showing a genome coverage of 6.62%. **(B)** Microbial read composition detected by mNGS. Among 2,036 microbial reads, 1,940 unique reads mapped to *C. burnetii*, accounting for 95.28% of all microbial reads. Low-level viral reads were also detected. **(C)** Targeted qPCR of *C. burnetii* nucleic acid. Blue curves indicate the patient’s blood samples, the pink curve indicates the positive control, and the green curve indicates the negative control. The amplification curves supported a positive result for *C. burnetii*. mNGS, metagenomic next-generation sequencing; qPCR, quantitative polymerase chain reaction; Rn, normalized reporter fluorescence.

On hospital day 8, peripheral-blood mNGS (PACEseq, Hugobiotech, Beijing, China) identified 1,940\u00B0*C. burnetii*-specific sequence reads, with a relative abundance of 95.28% (Figures 3A,B). Low-level Epstein–Barr virus (EBV; 72 specific reads) and cytomegalovirus (CMV; 1 specific read) signals were also detected. In view of the low sequence counts, the immunosuppressed host background, and the subsequent clinical course, these findings were interpreted as low-level viral reactivation or background detection rather than active coinfection. *C. burnetii*-specific antibody testing was positive, and targeted qPCR further supported *C. burnetii* nucleic acid detection (Figure 3C). Taken together, the clinical presentation, mNGS findings, and confirmatory results supported a diagnosis of acute Q fever with disseminated manifestations, presenting as sepsis with multisystem involvement of the hematopoietic system, liver, and serosal surfaces.

### Final diagnosis and treatment adjustment

Empirical antibiotics were discontinued, and targeted therapy with doxycycline (200 mg orally twice daily) was initiated based on clinical experience of the infectious disease center team. It was an individualized treatment choice for severe Q fever with multiple organ damage. Currently, there is no high-level evidence-based medical support for this. Given the combination of high fever, pancytopenia, and markedly elevated ferritin, hemophagocytic lymphohistiocytosis (HLH) could not be fully excluded at that time; a short course of methylprednisolone was added for immunomodulation, and eltrombopag (25 mg/day) was introduced to support platelet recovery. Supportive measures included intravenous albumin, diuretics, electrolyte correction, hepatoprotective agents, and nutritional support.

Echocardiography showed no valvular vegetations and only a trivial pericardial effusion; endocarditis was not identified, although surveillance for Q fever-related cardiac complications was maintained. Pleural fluid testing was negative for acid-fast bacilli and *M. tuberculosis* DNA, and blood cultures remained negative throughout hospitalization.

Body temperature normalized within 24 h of doxycycline initiation and remained normal thereafter. Abdominal distension, abdominal pain, and lower-limb edema progressively resolved, and mental status improved. On hospital day 12, the doxycycline dose was reduced to 100 mg twice daily. By hospital day 15, inflammatory markers had declined substantially, albumin had increased to 41.3 g/L, and PLT had risen to 102 × 10^9^/L; pleural and peritoneal effusions had largely resolved (Figures 2D–F). Aspirin was restarted for secondary cerebrovascular prevention. Hepatic enzyme normalization lagged behind other parameters but showed a consistent improving trend. By the later stages of hospitalization, he was ambulatory, with resolved gait instability and no fever, abdominal symptoms, or joint complaints.

### Follow-up and outcome

The patient was discharged on hospital day 18. Doxycycline 100 mg twice daily was continued for four additional days to complete a 14-day course. Aspirin, rosuvastatin, entecavir, and hepatoprotective therapy were maintained, and outpatient follow-up was arranged with the infectious disease, rheumatology, and neurology clinics.

At follow-up 20 days after discharge, WBC (4.33 × 10^9^/L), CRP (8.68 mg/L), and PLT (303 × 10^9^/L) had normalized, with no recurrent fever or other infectious symptoms. Mild anemia (Hb 92 g/L), markedly elevated erythrocyte sedimentation rate (ESR 90 mm/h; reference 0–15 mm/h), and mild cholestasis persisted; these findings were attributed clinically to ongoing RA disease activity rather than Q fever relapse or persistent infection. The patient entered a stable recovery phase, with management focused on control of the underlying rheumatologic disease.

## Discussion

*C. burnetii* infection has a wide clinical spectrum, but severe disease presenting as sepsis with multisystem involvement, as in this case, is uncommon. Most cases of acute Q fever are self-limited, with pneumonia and hepatitis among the more frequently described manifestations (1, 3). Severe forms with hematologic, hepatic, and serosal involvement have been reported mainly in patients with underlying immune dysfunction or immunosuppressive therapy. In this patient, long-term immunosuppressive therapy for RA may have contributed to impaired cell-mediated immunity and atypical dissemination of *C. burnetii*. The absence of a clear epidemiological exposure history, including denied animal contact and recent travel, further complicated early recognition. This feature is consistent with reports of Q fever in patients without obvious occupational or recreational animal exposure, possibly reflecting environmental aerosol transmission that can be difficult to trace retrospectively (18–21).

The diagnostic course illustrates the challenges of recognizing Q fever during severe acute infection. Blood cultures remained negative, consistent with the obligate intracellular nature of *C. burnetii* and its poor recovery on routine culture media. Serology is the standard diagnostic approach, but it was not pursued initially because of the patient’s acute deterioration and the need for timely pathogen identification. By hospital day 7, after several days of empirical antimicrobial therapy without clinical response and persistently negative conventional microbiological investigations, peripheral-blood mNGS was performed. Detection of 1,940\u00B0*C. burnetii*-specific reads at a relative abundance of 95.28% provided a clinically important diagnostic clue and allowed timely adjustment of antimicrobial therapy. Subsequent *C. burnetii*-specific antibody testing and targeted qPCR supported the mNGS result. This case suggests that, in selected immunocompromised patients with severe culture-negative sepsis of undetermined origin, earlier consideration of mNGS may help shorten the diagnostic interval and reduce exposure to ineffective empirical therapy (5, 6, 11, 20, 21).

The low-level EBV (72 reads) and CMV (1 read) signals detected by mNGS require cautious interpretation. In immunosuppressed patients, these viruses may be detected by sensitive molecular assays because of subclinical reactivation or background low-level viremia without overt end-organ disease (20, 22). In this patient, the very low read counts, absence of clinical or laboratory features suggesting active herpesvirus disease, and improvement after doxycycline-based therapy favored incidental viral reactivation or background detection rather than clinically significant coinfection. This distinction is important when interpreting mNGS results in immunosuppressed hosts, in whom low-level viral signals can otherwise be overattributed to the acute illness.

The combination of high fever, progressive pancytopenia, and markedly elevated ferritin raised concern for hemophagocytic lymphohistiocytosis (HLH) during the workup (23, 24). Bone marrow examination did not show hemophagocytosis, and no alternative HLH trigger other than *C. burnetii* infection was identified. The available findings were insufficient to establish HLH by established criteria, but the possibility could not be fully excluded when treatment decisions were made. A short course of methylprednisolone was therefore added as an immunomodulatory measure alongside doxycycline. The subsequent clinical improvement without HLH-directed escalation was compatible with *C. burnetii* infection as the primary driver of the hyperinflammatory state. Eltrombopag was used because of severe thrombocytopenia, but its contribution relative to infection control cannot be determined from a single case.

Doxycycline is the established first-line therapy for acute Q fever, with standard adult regimens generally using 100 mg twice daily for 14 days (1). In this patient, the treating team selected an initial higher dose of 200 mg twice daily in view of the severity of illness, including persistent high fever, sepsis with multisystem involvement, and progressive thrombocytopenia (25). The dose was reduced to the standard regimen on hospital day 12 after clinical stabilization. Defervescence within 24 h and subsequent improvement in laboratory and imaging findings were consistent with a favorable response to doxycycline-based therapy. However, this single case cannot determine whether the initial higher-dose regimen provided additional benefit over standard dosing, and evidence supporting higher-dose doxycycline in severe acute Q fever remains limited.

The concurrent right middle cerebral artery (MCA) occlusion with right temporal lobe infarction at admission should be considered separately from the *C. burnetii* infection. The cerebrovascular symptoms began several days before fever and systemic inflammation, with gait instability and dizziness as the initial complaints. Vascular imaging was consistent with ischemic infarction on a background of presumed atherosclerotic disease, supported by the 40-year smoking history. No clinical, echocardiographic, or laboratory findings, including the absence of valvular vegetations, suggested Q fever endocarditis or Q fever-related vascular pathology. The MCA occlusion was therefore considered more likely an independent concurrent cerebrovascular event rather than a manifestation of *C. burnetii* infection. This interpretation was reflected in management: aspirin was withheld during the acute thrombocytopenic phase and restarted for secondary cerebrovascular prevention after platelet recovery.

This report has several limitations. As a single case, the findings are not generalizable, and any causal inference regarding the contribution of immunosuppression to disease severity should remain cautious. Dynamic phase I and phase II serological data, which would have helped confirm the infection stage and support long-term assessment for chronic Q fever, were not available because of local laboratory constraints. The cost-effectiveness of early mNGS use in this setting was not assessed. Whether the initial higher-dose doxycycline regimen conferred benefit over standard dosing also remains uncertain. Prospective studies are needed to better define risk factors for severe disseminated Q fever in immunocompromised hosts and to clarify diagnostic and therapeutic strategies for this population.

Based on the results of this case, it can be concluded that in selected immunocompromised patients with severe infection and persistently negative conventional investigations, mNGS may serve as an adjunctive tool to facilitate timely pathogen identification and guide targeted antimicrobial therapy.

## Data Availability

The original contributions presented in the study are included in the article/Supplementary material, and further inquiries can be directed to the corresponding author.

## References

[1] Anderson A Bijlmer H Fournier PE Graves S Hartzell J Kersh GJ . Diagnosis and management of Q fever--United States, 2013: recommendations from CDC and the Q fever working group. MMWR Recomm Rep. (2013) 62:1–30. 23535757

[2] Eldin C Lenotte C Mediannikov O Ghigo E Million M Edouard S . From Q fever to *Coxiella burnetii* infection: a paradigm change. Clin Microbiol Rev. (2017) 30:115–90. doi: 10.1128/CMR.00045-16 27856520 PMC5217791

[3] Mezouar S Desnues B Bechah Y Bardin N Devaux C Mege JL. Pathogenicity and virulence of *Coxiella burnetii*: focus on Q fever. Virulence. (2025) 16:2495842. doi: 10.1080/21505594.2025.2495842, 40536474 PMC12184199

[4] Angelakis E Raoult D. Q fever. Vet Microbiol. (2010) 140:297–309. doi: 10.1016/j.vetmic.2009.07.016, 19875249

[5] Luo Y Zhao C Chen H Lin Z Xia J Liu X . Clinical value of metagenomic next-generation sequencing in diagnosis of *Coxiella burnetii* infection. BMC Infect Dis. (2025) 25:1219. doi: 10.1186/s12879-025-11581-3, 41023882 PMC12481755

[6] Sun L Yin Y Guo Y Chen H Wang H. Metagenomic next-generation sequencing enhances the diagnosis of Q fever: a retrospective observational study. Travel Med Infect Dis. (2025) 65:102845. doi: 10.1016/j.tmaid.2025.102845, 40169073

[7] Agostino T Edwards M. Q fever, an under-recognized and under-diagnosed disease: a case report. Cureus. (2024) 16:e70283. doi: 10.7759/cureus.70283, 39463649 PMC11512651

[8] Grey V Simos P Runnegar N Eisemann J. Case report: a missed case of chronic Q fever infective endocarditis demonstrating the ongoing diagnostic challenges. Access Microbiol. (2023) 5:000628.v3. doi: 10.1099/acmi.0.000628.v3 PMC10702382 38074103

[9] Van Roeden SE Wever PC Kampschreur LM Gruteke P Van Der Hoek W Hoepelman AIM . Chronic Q fever-related complications and mortality: data from a nationwide cohort. Clin Microbiol Infect. (2019) 25:1390–8. doi: 10.1016/j.cmi.2018.11.023, 30543852

[10] Fournier PE Raoult D. Comparison of Pcr and serology assays for early diagnosis of acute Q fever. J Clin Microbiol. (2003) 41:5094–8. doi: 10.1128/JCM.41.11.5094-5098.2003, 14605144 PMC262519

[11] Zhang X Chen H Han D Wu W. Clinical usefulness of metagenomic next-generation sequencing for Rickettsia and *Coxiella burnetii* diagnosis. Eur J Clin Microbiol Infect Dis. (2023) 42:681–9. doi: 10.1007/s10096-023-04586-w, 36997767 PMC10172222

[12] Chiu CY Miller SA. Clinical metagenomics. Nat Rev Genet. (2019) 20:341–55. doi: 10.1038/s41576-019-0113-7, 30918369 PMC6858796

[13] Watts GS Hurwitz BL. Metagenomic next-generation sequencing in clinical microbiology. Clin Microbiol Newsl. (2020) 42:53–9. doi: 10.1016/j.clinmicnews.2020.03.004

[14] Fan Z Mei M Chen C. Pyogenic liver abscess and sepsis caused by mixed anaerobic bacteria in an immunocompetent adult: a case report. Front Med. (2025) 12:1486256. doi: 10.3389/fmed.2025.1486256, 40018345 PMC11864932

[15] Li H Yu J Zeng Z Liu C Zhang Y Xia H . Clinical and pathological features of cerebrospinal meningitis caused by *Pantoea agglomerans*: a case report. BMC Infect Dis. (2024) 24:1150. doi: 10.1186/s12879-024-10035-6, 39396943 PMC11472509

[16] Liu D Li N Zhu Y Chen Q Fan X Feng J. Diagnosis of human angiostrongyliasis in a case of hydrocephalus using next-generation sequencing: a case report and literature review. BMC Neurol. (2024) 24:281. doi: 10.1186/s12883-024-03663-7, 39134956 PMC11318341

[17] Zhang H Liu Z Guan Y Li D Liu H Ruan L. Case report: metagenomics next-generation sequencing in the diagnosis of septic shock due to *Fusobacterium necrophorum* in a 6-year-old child. Front Cell Infect Microbiol. (2024) 14:1236630. doi: 10.3389/fcimb.2024.1236630, 38435306 PMC10904578

[18] Garibay CG Leker K Zahid A Park S. A rare presentation of Q fever endocarditis. J Investig Med High Impact Case Rep. (2025) 13:23247096251381158. doi: 10.1177/23247096251381158, 41431797 PMC12739083

[19] Habedank D Bublak A Habedank B. Q fever endocarditis of the tricuspid valve transmitted in an urban setting with no livestock exposure: case report. BMC Infect Dis. (2024) 24:766. doi: 10.1186/s12879-024-09629-x, 39090536 PMC11293036

[20] Zhai Y. Q fever represented as multiple pulmonary nodules: a case report. J Int Med Res. (2023) 51:3000605231183553. doi: 10.1177/03000605231183553, 37382236 PMC10328026

[21] Zhan S Jin H Ji H Hou X Li J Zhang Y . Clinical diagnosis of Q fever by targeted next-generation sequencing for identification of *Coxiella burnetii*. BMC Infect Dis. (2025) 25:190. doi: 10.1186/s12879-024-10437-6, 39920575 PMC11806902

[22] Speth P Jargosch M Seiringer P Schwamborn K Bauer T Scheerer C . Immunocompromised patients with therapy-refractory chronic skin diseases show reactivation of latent Epstein–Barr virus and cytomegalovirus infection. J Invest Dermatol. (2022) 142:549–558.e6. doi: 10.1016/j.jid.2021.07.171 34480891

[23] Henter JI. Hemophagocytic Lymphohistiocytosis. N Engl J Med. (2025) 392:584–98. doi: 10.1056/NEJMra2314005, 39908433

[24] Henter JI Horne A Aric M Egeler RM Filipovich AH Imashuku S . HLH-2004: diagnostic and therapeutic guidelines for hemophagocytic lymphohistiocytosis. Pediatr Blood Cancer. (2007) 48:124–31. doi: 10.1002/pbc.21039 16937360

[25] Hoofnagle JH. "Doxycycline". In: LiverTox®: Clinical and Research Information on Drug-Induced Liver Injury. Bethesda, MD: National Institute of Diabetes and Digestive and Kidney Diseases (2012) 31643176

