# Supplementary material for: Acute Coxiella burnetii infection presenting as sepsis with multisystem involvement in an immunosuppressed patient: a case report
Source: Front Med (Lausanne). 2026 Aug 24;13:1899347. doi: 10.3389/fmed.2026.1899347 (PMC13547048; doi:10.3389/fmed.2026.1899347)
Supplement: Supplementary file 1 [file Supplementary_file_1.DOCX]

**Supplementary Table 1. Serial laboratory findings during hospitalization and follow-up**

|  | **Time point** | | | | | | | |
| --- | --- | --- | --- | --- | --- | --- | --- | --- |
| **Laboratory parameters** | **Day 1** | **Day 2** | **Day 5** | **Day 7** | **Day 11** | **Day 15** | **Day 18** | **Day 38** |
| **WBC (3.5–9.5 × 10⁹/L)** | 2.18 | 2.57 | 3.31 | 3.91 | 4.95 | 4.33 | 3.46 | 4.33 |
| **PLT (125–350 × 10⁹/L)** | 88 | 66 | 41 | 45 | 32 | 102 | 104 | 303 |
| **Hb (130–175 g/L)** | 98 | 87 | 95 | 83 | 76 | 84 | 76 | 92 |
| **PCT (<0.05 ng/mL)** | 1.01 |  | 1.25 |  | 0.53 | 0.31 |  |  |
| **CRP (0–10 mg/L)** | 54.75 | 61.16 | 74.86 | 67.14 | 45.17 | 20.61 | 12.66 | 8.68 |
| **GGT (10–60 U/L)** | 80.6 |  | 105.4 | 170.9 | 235.4 | 236.7 |  | 66.1 |
| **TBIL (<23 μmol/L)** | 12.25 |  | 20.19 | 37.95 | 39.23 | 26.9 |  | 16.1 |
| **DBIL (1.7–6.8 μmol/L)** | 6.02 |  | 13.16 | 28.74 | 25.81 | 17.27 |  | 8.04 |
| **Alb (40–55 g/L)** | 33.9 |  | 27.3 | 26.1 | 36.6 | 41.3 |  | 39.7 |
| **ALP (45–125 U/L)** | 117.4 |  | 208.1 | 317.3 | 307.4 | 349.7 |  | 127.4 |
| **AST (15–40 U/L)** | 100 | 79.6 | 74.5 | 85.8 | 97 | 82.2 |  | 41.6 |
| **ALT (9–50 U/L)** | 79.9 |  | 58.6 | 56.2 | 69.5 | 64.3 |  | 33.1 |

**Metagenomic next-generation sequencing and bioinformatic analysis**

DNA was extracted using the Nucleic Acid Extraction Reagent Kit (YGZZ014, HugoBiotech, Beijing, China), and sequencing libraries were constructed using the Universal Sequencing Reaction Kit (YGZZ005, HugoBiotech, Beijing, China). The concentration and integrity of the libraries were measured using Qubit 4.0 (Thermo Fisher Scientific, USA) and Agilent A2100 Bioanalyzer (Agilent Technologies, USA). After quality control, sequencing was performed on the Illumina 550DX platform (Illumina, Inc, USA) with single-end 75 bp reads. For each batch of experiment, a no-template control (NTC) was set up, taking sterile water as the initial input.

After obtaining the raw data, bcl2fastq (v2.20.0.422) with default parameters was employed to convert the “bcl” files into fastq format and to split data of each sample in a batch. Then, fastp (v0.24.0) [1], with its default parameters, was used to filter out adapters, low-quality sequences and sequences with high percentage of “N”, thus generating high quality sequences with a minimum length greater than 50 bp. Next, BWA (0.7.15) [2] of “mem” mode was used to align the sequences to the human genome (GRCh38.101), thereby removing host-derived sequences. The remaining reads were then aligned to an in-house microbial genome database using bwa, which was compiled from NCBI Refseq and GenBank databases. Subsequently, microbial identification was carried out based on the alignment results. The number of unique aligned reads and RPM (reads per million) for each identified microorganism were calculated. Positive mNGS results were defined according to the following criteria: For the detected bacteria (*Mycobacterium* excluded), fungi (*Cryptococcus* excluded), and parasites, the positive criteria for the mNGS result were set as follows: (1) genome coverage of the unique reads mapped to this microorganism ranked top10 of the same kind of microbes and the microorganism was not detected in the NTC; or (2) RPM_sample_/RPM_NTC_ was > 10 (RPM_NTC_ ≠ 0). For viruses, *Mycobacterium tuberculosis*, and *Cryptococcus*, a positive mNGS result was considered when it was not detected in NTC and at least 1 unique read was mapped to species or when RPM_sample_/RPM_NTC_ was > 5 (RPM_NTC_ ≠ 0).

**Reference**

1.	Chen, S., et al., fastp: an ultra-fast all-in-one FASTQ preprocessor. Bioinformatics, 2018. 34(17): p. i884-i890.

2.	Li, H. and R. Durbin, Fast and accurate long-read alignment with Burrows–Wheeler transform. Bioinformatics, 2010. 26(5): p. 589-595.
